# 

**Published:** 1852-12

**Authors:** 


### CONTENTS

### OF NO VI.

---

### ORIGINAL COMMUNICATIONS.

### ESSAYS AND CASES.

ART. I.—Laryngismus Stridulus. By J. W. BRIGHT, M. D., of Louisville, Ky., - - - - - - - - 462

ART. II.—A case of Stone in the Bladder, at Astoria, Oregon. By ISRAEL MOSES, M. D., Surgeon U. S. A., - - 462

ART. III.—A case of Hematemesis. By Dr. G. M. BENNET, of Smithsville, Indiana. - - - - - - - 471

### SELECTIONS FROM FOREIGN AND HOME JOURNALS

On variations of Pitch in Percussion and Respiratory Sounds, and their application to Physical Diagnosis. - - - 475

Practice in Philadelphia. - - - - - - - - 531

Friendly Injury - - - - - - - - - - 532

Mineral Springs - - - - - - - - - 532

On the use of Manganese as an Adjuvant to Iron - - - 533

On the Internal Administration of Chloroform in Delirium Tremens - - - - - - - - - - 535

Diarrhœa successfully Treated by Sulphuric Acid. - - - 538

On the Proportion of the Subjects bitten by Mad Animals, who become affected with Hydrophobia. - - - - - - 539
On the Induction of Abortion in the Vomiting of Pregnant Women. - - - - - - - - - - - 542
On a case of Poisoning from Swallowing Chloroform, and on its Administration in Lead Colic. - - - - - - 545

ORIGINAL INTELLIGENCE.

University of Louisville - - - - - - - - - 547
Our next Volume - - - - - - - - 548
Prof. Flint's Prize Essay. - - - - - - - - - 548
Death of Dr. Drake - - - - - - - - - - 548
Books Received. - - - - - - - - - 552
Prize Essays for 1853. - - - - - - - - 552

